# Unspoken Assumptions in Multi-layer Modularity maximization

**DOI:** 10.1038/s41598-020-66956-0

**Published:** 2020-07-06

**Authors:** Obaida Hanteer, Matteo Magnani

**Affiliations:** 1 IT University of Copenhagen, Copenhagen, 2300 Denmark; 2 InfoLab, Department of Information Technology, Uppsala University, Uppsala, Sweden

**Keywords:** Applied mathematics, Computational science, Computer science

## Abstract

A principled approach to recover communities in social networks is to find a clustering of the network nodes into modules (i.e groups of nodes) for which the modularity over the network is maximal. This guarantees partitioning the network nodes into sparsely connected groups of densely connected nodes. A popular extension of modularity has been proposed in the literature so it applies to multi-layer networks, that is, networks that model different types/aspects of interactions among a set of actors. In this extension, a new parameter, the coupling strength *ω*, has been introduced to couple different copies (i.e nodes) of the same actor with specific weights across different layers. This allows two nodes that refer to the same actor to reward the modularity score with an amount proportional to *ω* when they appear in the same community. While this extension seems to provide an effective tool to detect communities in multi-layer networks, it is not always clear what kind of communities maximising the generalised modularity can identify in multi-layer networks and whether these communities are inclusive to all possible community structures possible to exist in multi-layer networks. In addition, it has not been thoroughly investigated yet how to interpret *ω* in real-world scenarios, and whether a proper tuning of *ω*, if exists, is enough to guarantee an accurate recoverability for different types of multi-layer community structures. In this article, we report the different ways used in the literature to tune *ω*. We analyse different community structures that can be recovered by maximising the generalised modularity in relation to *ω*. We propose different models for multi-layer communities in multiplex and time-dependent networks and test if they are recoverable by modularity-maximization community detection methods under any assignment of *ω*. Our main finding is that only few simple models of multi-layer communities in multiplex and time-dependent networks are recoverable by modularity maximisation methods while more complex models are not accurately recoverable under any assignment of *ω*.

## Introduction

Community detection is one of the core tasks in the analysis of complex networks. It involves partitioning the network into groups of nodes, also known as communities, partitions, cohesive groups or clusters. The importance of this task comes from its ability to break large networks into smaller building blocks so it becomes easier to understand the structure of the network and the role played by each node. Applications of community detection algorithms are ubiquitous, including social media group detection^1^, thematic community detection^2^, second-order flow analysis in human mobility^3^, and topic detection in information networks^4^. A community detection solution is usually represented as a clustering ${\mathcal{C}}$ where ${\mathcal{C}}$ = $\{{C}_{1},{C}_{2},\ldots ,{C}_{k}\}$, and *C*_1_, *C*_2_, *C*_*k*_ are disjoint or non-disjoint groups of nodes.

Although there is no agreed-upon definition for communities in networks, it is widely accepted to consider a community, loosely speaking, the group of nodes that are more densely connected with each other than with the rest of the network^5^. This definition is aligned with the quality function modularity^6,7^, which is a function *f* (${\mathcal{C}}$) that produces values in the range [−1, 1] and for a given clustering ${\mathcal{C}}$ over a network, it measures the extent to which the network nodes are more densely connected within the communities $\{{C}_{1},{C}_{2},\ldots ,{C}_{k}\}$ ∈ ${\mathcal{C}}$ than across these communities. While modularity was firstly proposed as a quality measure to evaluate the accuracy of community detection methods at the time^6^, it gave birth to a new class of community detection methods that interpreted the community detection problem into finding a clustering with the maximal modularity over the network.

In response to the advances on complex network analysis, the multi-layer network model has been proposed as an effective tool to consider different types and/or different time-windows of interactions in a given system^8,9^. The intuition behind such generalisation is that interactions among a set of actors do not happen in isolation. Instead, they inherit certain dependencies among each other such that one might cause the other one, or one can be used as predictor for the other one. For example, if two people are friends on Facebook, the probability of having those two connected via other means of communication (i.e., Twitter, Whatsapp, etc) is much higher than if they were not connected via any communication mean. Hence, studying these interactions separately without accounting for any dependencies among them might yield into misleading or incomplete findings.

**Figure 1:**
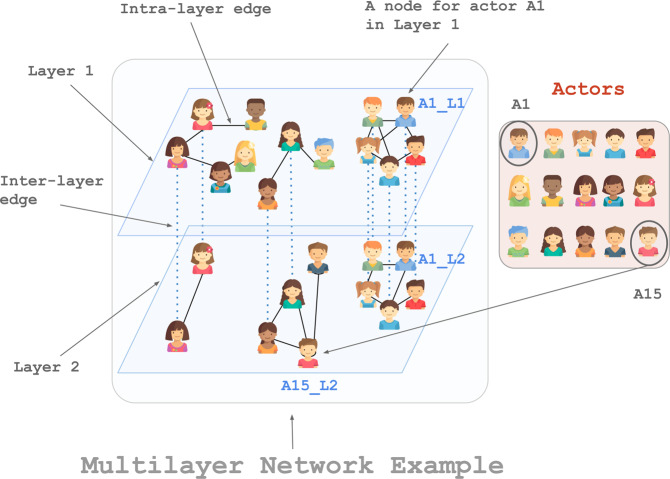
A schematic network with two types of interactions represented by Layer 1 and Layer 2 among 15 actors. The two nodes existing in both layers and representing the same actor (e.g. the same person) are linked by a dotted line.

Two special applications of the multi-layer model that received a great deal of attention in the literature are *multiplex* networks – that is, networks that model different types of relationships among a set of actors^10^, and *time-dependent* (or temporal) networks – that is, networks in which the interactions among actors change over time^11^. When the multi-layer model is used to model the former, layers correspond to different types of relationships and are thus categorical, while in the latter, layers represent different time-points of a relationship, and thus are ordinal. Multiplex networks have been used in a variety of different applications including, to mention a few, ecological networks (which model different types of interactions among species)^12^, and social networks (where different layers can be used to model different types of interactions provided by a social media platform or different relationships across different social media platforms)^13^. Temporal networks have been used to model brain network dynamics (where layers capture the interaction among different areas in the brain over time)^14^, and citation networks (where layers refer to different time points in which the citation happened)^15^. Figure 1 shows a typical layered representation of a multiplex network where each of the layers corresponds to a different type of interaction, and nodes in different layers can be associated to the same actor (the same person for example). The same figure can also be seen as a time-dependent network if layers were to refer to time-windows of an interaction instead. Here, we adopt the term *actor* from the field of social network analysis, where multiplex networks and time-dependent networks have been first applied, and the term *layer* from recent generalisations of these models^8^. We also refer to networks that model a single relationship among a set of actors as *single-layer* networks.

In spite of the plethora of methods developed for detecting communities in multi-layer networks, there is very little work on proposing a definition for multi-layer communities like community definitions used with single-layer networks. For example, it is common to accept the conceptualisation of communities based on the relative frequency of edges within and across the communities in single-layer networks, but no extension of this definition is provided in the literature for multi-layer networks. This can be due to two reasons: First, multi-layer community discovery as a tool emerged as a response to the generalisation of graphs into multi-layer graphs, and not to the generalisation of communities into multi-layer communities. Second, most multi-layer community detection methods are mathematical extensions of single-layer community discovery methods, which made it possible to provide such methods without having to investigate a precise extension of their original conceptualisations. From a structural perspective, recent work^16^ argues that there are two primary differences between a single-layer community and a multi-layer one. First, a multi-layer community can expand over multiple layers. Second, edges of a multi-layer community in one layer might depend on the connectivity patterns in another layer. For example, in a multiplex network that models different Twitter interactions among a set of users (i.e., following and retweeting each represented as a layer in a 2-layer multiplex network), it might be the case that the re-tweet edges among a set of actors are largely dependent on whether these actors follow each other or not, which might explain the existence of multi-layer communities that expand over the two layers.

Undoubtedly, the generalisation proposed by the multi-layer framework has introduced new challenges to the community detection problem. While some researchers worked on that by tailoring new community detection methods for multi-layer networks^17^, there has been more tendency to extend some of the already existent methods used with single-layer networks like collapsing the multiple layers into a single-layer network and then use any of the single-layer community detection methods^18^, extended label propagation^19^ and extended clique percolation^20^ just to mention few.

A popular extension from single-layer to multi-layer is the extension of the quality function *modularity* into the *generalised modularity*^21^. In that extension, the authors introduce a new parameter, the coupling strength *ω*, to the modularity function. The new proposed parameter, *ω*, assumes that nodes of the same actor in different layers are coupled via coupling edges and these coupling edges are weighted with an amount equals to *ω*. With that extension, a modularity maximisation method does not only maximise the within-community intra-layer edges and minimise the cross-community intra-layer edges, but also maximises the sum of coupling edges weights, i.e., nodes that belong to the same actor, within a community given *ω*. Indeed, with *ω* being the only multi-layer ingredient in the generalised modularity formula, there has been few tries in the literature to tune *ω* such that it reflects some of the inter-layer information across different layers (i.e., closeness between layers, common neighbour across layers, etc.). It is not clear, however, how these different interpretations affect multi-layer community detection using modularity maximisation and whether the modularity function responds to these interpretations by providing clusterings that support the intuition behind them. For example, the intuition behind tuning *ω* such that it reflects the percentage of common neighbours among nodes in different layers is that it makes more sense for multi-layer communities to group nodes of the same actor in one community only if they have similar neighbourhoods across the layers^22^. The question is whether modularity responds to that tuning by clustering the network nodes into communities that respect that intuition.

**Figure 2:**
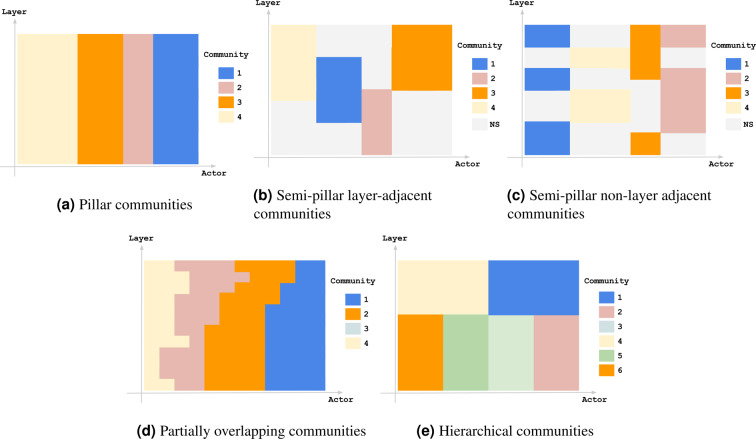
Different models of multi-layer communities, illustrated using colors referring to the actors’ community memberships across different layers. NS in (**b,c**) means that the community membership of the actors in these layers is not specified in this illustration as it does not expand over multiple layers. (**a**) Pillar communities. (**b**) Semi-pillar layer-adjacent communities. (**c**) Semi-pillar non-layer adjacent communities. (**d**) Partially overlapping communities. (**e**) Hierarchical communities.

We claim that methods that maximise the generalised modularity in multi-layer networks inherited their popularity and success from the modularity maximisation methods applied to single-layer networks before, yet the communities identified by these methods are not thoughtfully investigated enough. For example, Fig. 2 illustrates multi-layer communities that can exist in multi-layer networks as we will discuss later. We ask the question whether these different models of multi-layer communities are recoverable by maximising the generalised modularity under any assignment of *ω*.

The goal of this article is to analyse the assumptions behind the generalised modularity and the effect of that on the possible community structures that can be recovered in multi-layer networks as a result of maximising that quality function. To achieve that, we first propose different models for multi-layer communities in multiplex and time-dependent networks by hypothesising some real-world scenarios. We then analyse different community structures that can be recovered by maximising the generalised modularity in relation to *ω*. We conclude from our experiments that only few simple models of the multi-layer communities we propose are recoverable by modularity maximisation methods while more complex models are not accurately recoverable under any tuning for *ω*.

The novelty of this article comes in **(1)** the community models it proposes to model different community structures which can exist in multi-layer networks, and **(2)** the discussion about the limitations of the highly popular and extensively referenced methods based on modularity maximisation for recovering communities in multi-layer networks. Unless mentioned otherwise, we will refer to the generalised modularity as modularity, and to the modularity applicable only on single-layer networks as single-layer modularity.

Our article is structured as follows. We first start by proposing different models for multi-layer communities possible to exist in multiplex and time-dependent networks in Section 1. Since understanding the generalised modularity requires a very good understanding of single-layer modularity, we first recall the definition of modularity in single-layer networks (Section 2). This is followed by a description of generalised modularity in Section 3. We study the effect of *ω* on the types of multi-layer communities that can be recovered by maximising modularity in Section 4. We report the results of our experiments on the recoverability of our proposed community models using modularity maximisation in Section 5. We discuss our findings in Section 6, and report the methods used for our experiments in Section 7.

## Models for Multi-layer Communities in Multiplex and Time-dependent Networks

Despite the variety of existing generative models for multi-layer communities in multi-layer networks^16,23–31^, they focus on a limited amount of structures for multi-layer communities, or produce multi-layer communities that are not straightforwardly mappable to real-world scenarios in multi-layer networks. Here we hypothesize some real-world scenarios for multi-layer communities and interpret them into different multi-layer community models. These models can exist in multiplex and time-dependent networks under different conditions. While we do not claim that this list of models is exhaustive to all possible communities that can exist in multi-layer networks, we claim that they provide a good categorization for different types of multi-layer communities for future work to investigate further generalisations. We refer to the nodes of an actor *a* in different layers with a non-zero degree in their layers as the *active nodes* of *a*. For a multi-layer community *C* that expands over multiple layers, we refer to the set of nodes of *C* in a single layer *L* as the induced nodes of *C* in *L*, and to the set of actors resulted by mapping each of the induced nodes of *C* in a layer *L* to their actors as the induced actors of *C* in *L*.

- **[M1] Pillar communities**: We call a multi-layer community *C* a pillar community if there exists a set of actors ${\mathcal{A}}=\{{a}_{1},{a}_{2}\mathrm{,..}.{a}_{k}\}$ such that *C* is constituted of only the active nodes of all the actors in ${\mathcal{A}}$ in all layers of the multi-layer network. Pillar communities result from a very high dependency among actors connectivity patterns on all layers of the network, which results in an aligned grouping of the relevant nodes in each layer.
- **[M2] Semi-pillar layer-adjacent communities**: We call a multi-layer community *C* a semi-pillar layer-adjacent community if there exists a set of actors ${\mathcal{A}}=\{{a}_{1},{a}_{2},\,\mathrm{..}.{a}_{k}\}$ such that *C* is constituted of only the active nodes of all the actors in ${\mathcal{A}}$ in a subset of layers of the multi-layer network and these layers are adjacent to each other. Semi-pillar layer-adjacent communities usually evolve in time-dependent networks where layers refer to specific time-windows. In these networks, a set of actors might engage in the same community for a limited time and then disappear or engage in other groups in subsequent time-windows. This might result in semi-pillar communities that expand over a subset of the layers that are adjacent to each other.
- **[M3] Semi-pillar non-layer-adjacent communities**: Similar to [M2] except that the layers where the community expands are not adjacent to each other. These communities might evolve in multiplex networks where layers do not necessarily have an order and the layers where the semi-pillar communities expand are not adjacent to each other. These communities also might evolve in time-dependent networks if a group of actors engage in a community for a couple of consecutive time-windows then disappear or engage in other groups in subsequent time-windows, then engage again in the same community.
- **[M4] Partially overlapping communities**: A multi-layer community $C$ that expands over multiple layers is partially overlapping if the sets of the induced actors of $C$ in each layer where the community expands partially (but not completely) overlap. These communities evolve in cases when the community membership of a set of actors in one layer *l*_1_ influences the community membership of only a subset of these actors in another layer *l*_2_ while the membership of the rest of these actors in *l*_2_ does not necessarily depend on their membership in *l*_1_. Think of an example where the network is a three-layer multiplex network modelling Twitter interactions (following, retweeting and replying) among a set of actors. It might be the case that the community membership of a set of actors in the ‘following’ layer influences the community membership of only a subset of these actors in the ‘retweet’ or ‘reply’ layers (i.e., user ${a}_{1}$ retweets user ${a}_{2}$ because they follow each other) while the community membership of the rest of these actors on these layers does not really depend on the ‘following’ layer.
- **[M5] Hierarchical communities**: A multi-layer community $C$ that expands over multiple layers is hierarchical if there is a hierarchy among the sets of the induced actors of $C$ in the layers where it expands. A hierarchical community evolves when the grouping of a set of actors in a layer ${L}_{1}$ still depends on the community membership of these actors in another layer ${L}_{2}$ but additional non-layer specific dependencies might result in different divisions of this grouping across the layers which breaks the perfect cross-layer group alignment that happens in the pillar model. Think of the 3-layer multiplex modelling Twitter interactions mentioned above. A grouping of a set of actors in the retweeting’ layer might still depend on whether they follow each other or not (i.e., user ${a}_{1}$ retweets user ${a}_{2}$ only if they follow each other), but some other user-specific properties (political affiliation for example) might result in multiple divisions of their groupings across the two layers.

Figure 2 provides an illustration for the different multi-layer community models defined above.

## Modularity in Single-layer Networks

As firstly proposed by^6,7^, the modularity of a clustering ${\mathcal{C}}$ over a single-layer network characterised by an adjacency matrix $A$, where ${A}_{(i,j)}$ is 1 if there is an edge between nodes i and j and zero otherwise, can be written as:

1 $$Q=\frac{1}{2|E|}\sum _{C\in {\mathscr{C}}}\sum _{(i,j)\in C}[{A}_{(i,j)}-{P}_{(i,j)}]$$

where $|E|$ is the number of edges in the network. The summation is performed only over pairs of nodes that belong to the same community *C* ∈ ${\mathcal{C}}$. ${P}_{(i,j)}$ is the probability of having an edge between nodes i and j in a null model.

The intuition behind modularity is to measure the extent to which the distribution of edges in a network is far from what one would expect shall the edges be distributed in a community-less manner. It can be seen as a normalised distance between a network $N$ that has a specific edge distribution and an equivalent network, usually referred to as the null model, where edges are distributed randomly. Two common choices for the null model are: the uniform random model, where the probability of having an edge among any pair of nodes in the network is fixed, or the non-uniform random model, where the probability of having an edge among two nodes depends on their degrees (also called a null model with preferential attachment dynamic).

Modularity assumes that nodes within a community tend to interact more densely with each other than with the rest of the network. Hence, the quality of a community, from a modularity perspective, is in the percentage of within-community edges out of all edges incident to nodes of that community. With that being said, we stress out the fact that modularity is not a property of the network, but rather a property of a clustering over the network. It is common, however, to describe a network as modular and that is to refer to the existence of a significantly modular clustering over that network. The modularity of a clustering ${\mathcal{C}}$ in a network can be translated into calculating the normalised sum of the network edges contributions to ${\mathcal{C}}$. While iterating through network edges, some edges come as a surprise (they exist in the network, but not highly probable to appear in the null model), and others are expected (highly probable in the null model). Surprising edges contribute more to the final score when they happen to be within a community. Equation 1 therefore guarantees:

- [**P1**] Rewarding existent edges within communities. Each edge connecting two nodes i and j in a community *C*_*x*_ ∈ ${\mathcal{C}}$ contributes positively to the total sum with an amount equal to the difference $\mathrm{(1}-{P}_{i,j})$. Clearly, edges that come as a surprise, i.e., are not expected in the null model and hence result in a very low value of ${P}_{i,j}$, contribute more to the final modularity than the expected ones.
- [**P2**] Punishing non-existent edges within communities. If two nodes i, j happened to be in the same community *C*_*x*_ ∈ ${\mathcal{C}}$ and they are not directly connected (i.e.,A_i,j_ = 0), this contributes negatively to the modularity score. The negative contribution equals $(\,-\,{P}_{i,j})$. Meaning that highly expected edges contribute more negatively when they are absent than the lowly expected ones.
- [**P3**] Punishing existing edges among communities. Even though it is not straightforward to see that in the equation, clusterings with less cross-community edges score higher modularity than those with more cross-community edges. The reason is that modularity counts only the contribution of edges lying within communities. The existence of edges among two communities will result in a larger number of edges in the network (larger $2|E|$ in the equation) and zero contribution to the modularity score. This will result in a lower modularity than if edges did not exist across communities.
- [**P4**] Rewarding non-existing edges among communities. Following the same reasoning discussed in P3, non-existent edges among communities means smaller $|E|$ which results in a higher modularity.

**Figure 3:**
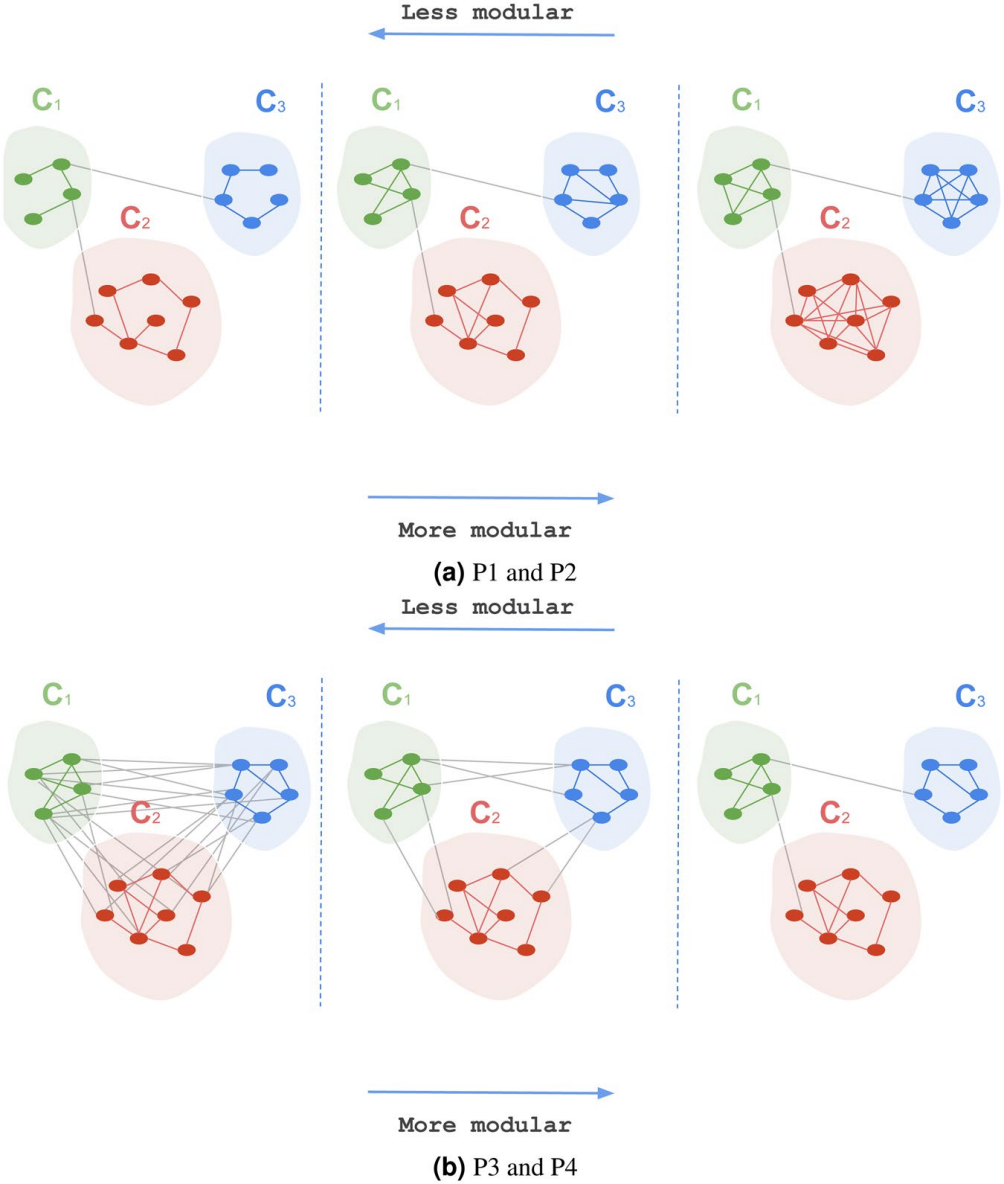
The effect of within-community and cross-community edges on the modularity score of the clustering ${\mathcal{C}}$ = {*C*_1,_ *C*_2,_ *C*_3_} over the single-layer network illustrated in figures (**a**,**b**). (**a**) P1 and P2, (**b**) P3 and P4.

Figure 3 illustrates the effect of within-community and cross-community edges on the modularity score.

## From Single-layer Modularity to Generalised Modularity

**Figure 4:**
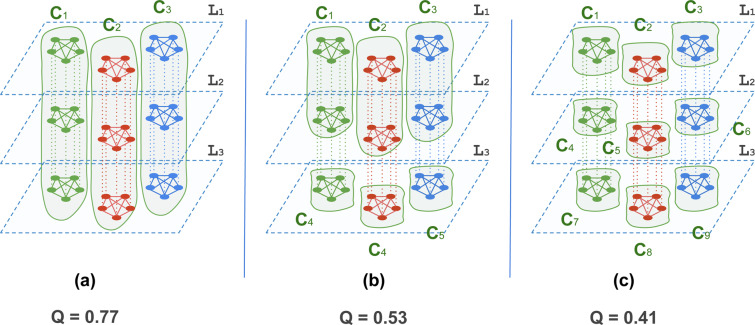
Modularity scores, calculated using multi-layer modularity, for three different clusterings over the same multiplex network constituted of 3 layers and 15 actors and edges that are distributed across three cliques per layer. (**a**) Q = 0.77, (**b**) Q = 0.53 (**c**) Q = 0.41.

A generalisation of modularity has been proposed for multi-layer networks^21^. In this generalisation, the authors introduce a new parameter $\omega$, also called the coupling strength, which is a weight assigned to the coupling edges connecting nodes of an actor across different layers. On the layer level, this parameter has been interpreted as the closeness among different layers^32^. On the actor level, it has been claimed that this parameter should reflect information about the extent to which an actor tends to have the same behaviour across layers (high $\omega$) or a different behaviour in different layers (low $\omega$)^22^. With $\omega$, the modularity score does not only reward existent within-community intra-layer edges and absent cross-community intra-layer edges, and penalise the absent within-community intra-layer edges and existent cross-community intra-layer edges in each layer, but also rewards the coupling edges (i.e., inter-layer edges) within communities with an amount proportional to $\omega$. This means that if two nodes ${n}_{ix}$, ${n}_{iy}$ which refer to the same actor i, and hence are coupled, happen to appear in the same community, this contributes to the multi-layer modularity score with an amount proportional to ${\omega }_{xy}$, that is the coupling strength assigned to the coupling edge between ${n}_{ix}$, ${n}_{iy}$. With that being said, the result of maximising modularity in multi-layer networks is not necessarily a clustering that groups all the nodes of an actor in one community. There are two forces that drive the partitioning in multi-layer networks using modularity maximisation. The first tries to keep the node in its optimal single-layer modularity grouping, and the second tries to group the node together with other nodes that refer to the same actor. To have an idea about modular structures in multi-layer networks according to multi-layer modularity, we report the multi-layer modularity scores (Fig. 4) of three different clusterings over a schematic multiplex network constituted of 3 layers and 15 actors and three cliques in each layer. The figure shows that even though the three clusterings equally optimise the distribution of intra-layer edges within and across the communities, the generalised modularity favors clusterings that maximise the coupling edges within communities in addition (Fig. 4a).

The generalised form of Eq. 1 as proposed by^21^ can be written as:

2 $$Q=\frac{1}{2\mu }\mathop{\underbrace{(\sum _{C\in {\mathcal{C}}}\sum _{({i}_{s},{j}_{s})\in C}[{A}_{(i,j)}^{s}-{P}_{(i,j)}])}}\limits_{{\rm{part1}}}+\frac{1}{2\mu }\mathop{\underbrace{(\sum _{C\in {\mathcal{C}}}\sum _{({i}_{s},{j}_{r})\in C}[{W}_{i,j}^{s,r}\ast \delta ({i}_{s},{j}_{r})])}}\limits_{{\rm{part2}}}$$

where ${A}_{i,j}^{s}$ is the adjacency matrix of layer $s$, ${W}^{s,r}$ is the coupling matrix that describes inter-layer edges between layers s, r^1^ and ${W}_{i,j}^{s,r}$ therefore equals the value of the coupling strength ${\omega }_{i,j}$ assigned to the coupling edge connecting nodes i from layer s and j from layer r. $\delta ({i}_{s},{j}_{r})$ is the Kronecker delta: it equals 1 if nodes ${i}_{s}$ and ${j}_{r}$ refer to the same actor otherwise it equals 0. $\mu$ is a normalisation factor and it equals the sum ${\sum }_{s}2|{E}_{s}|$ + the total possible number of inter-layer edges in the multi-layer network given the coupling type (complete or adjacent) and assuming that all actors are existent in all layers.

The first part of Eq. 2 is the same used to calculate single-layer modularity in Eq. 1. This part alone reaches its maximum when nodes in each layer are grouped according to their optimal single-layer modularity. The second part of this equation is the added multi-layer ingredient to the modularity score. This part alone is maximised when the single-layer optimised groupings are cross-merged across the layers such that all the overlapping single-layer groupings appear together in the same multi-layer community. The main difference between the two parts is that the first part penalises any other grouping of the nodes that does not respect the optimal single-layer modularity grouping, while the second part (assuming $\omega \, > \,0$) does not penalise but only rewards the co-existence of the same actor nodes in one community. If the contribution of the second part of Eq. 2 is small (i.e., $\omega$ is small), optimising modularity will keep nodes in each layer grouped according to their optimal single-layer modularities so the first part of Eq. 2 is optimised to its maximum value. At the same time if the contribution added by the second part is big enough (i.e., $\omega$ is large), this might not guarantee that nodes in each layer will be grouped according to their optimal single-layer modularity as the contribution of the coupling edges added to the total sum might become big enough to compensate for the penalties resulted by grouping nodes not according to their optimal single-layer modularity.

**Figure 5:**
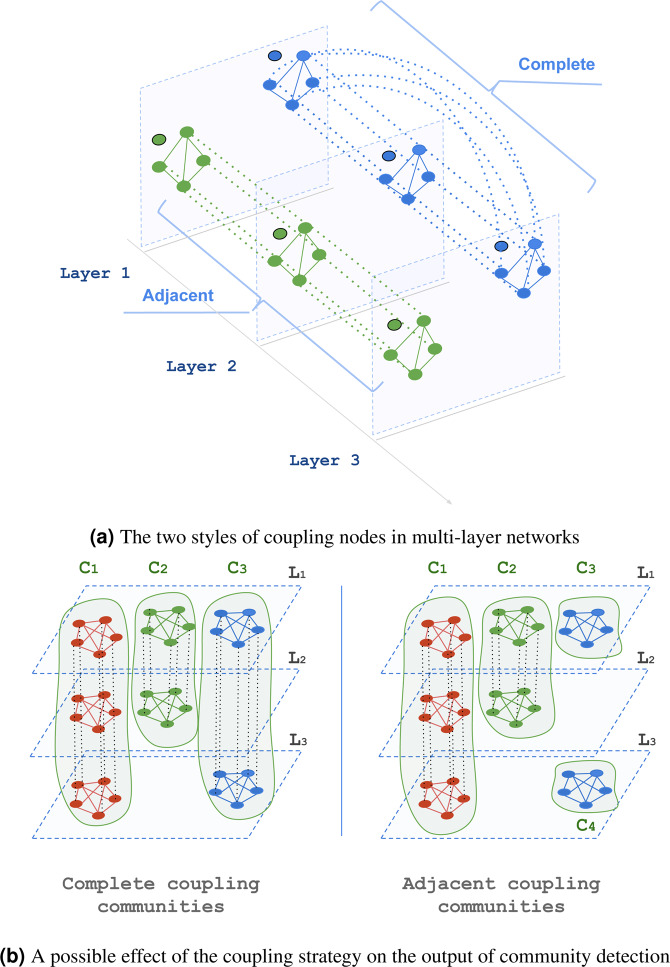
Styles of coupling nodes in multi-layer networks. (**a**) The two styles of coupling nodes in multi-layer networks. (**b**) A possible effect of the coupling strategy on the output of community detection.

The type of coupling chosen to connect nodes of the same actor across layers is of great importance for studying any dynamic in multi-layer networks especially for community detection using modularity maximisation. When layers are *ordinal*, i.e., they have a specific order like in the case of time-dependent networks, coupling edges connect each layer s with the successive layer s + 1 and the previous layer s-1. When layers are *categorical*, i.e., they do not follow a specific order like the case of multiplex networks, coupling edges connect all pairs of layers. We refer to these two types of couplings as adjacent coupling, and complete coupling for time-dependent and multiplex networks respectively. Figure 5 illustrates the two different types of coupling strategies and the effect of that on a process like community detection in multi-layer networks.

**Figure 6:**
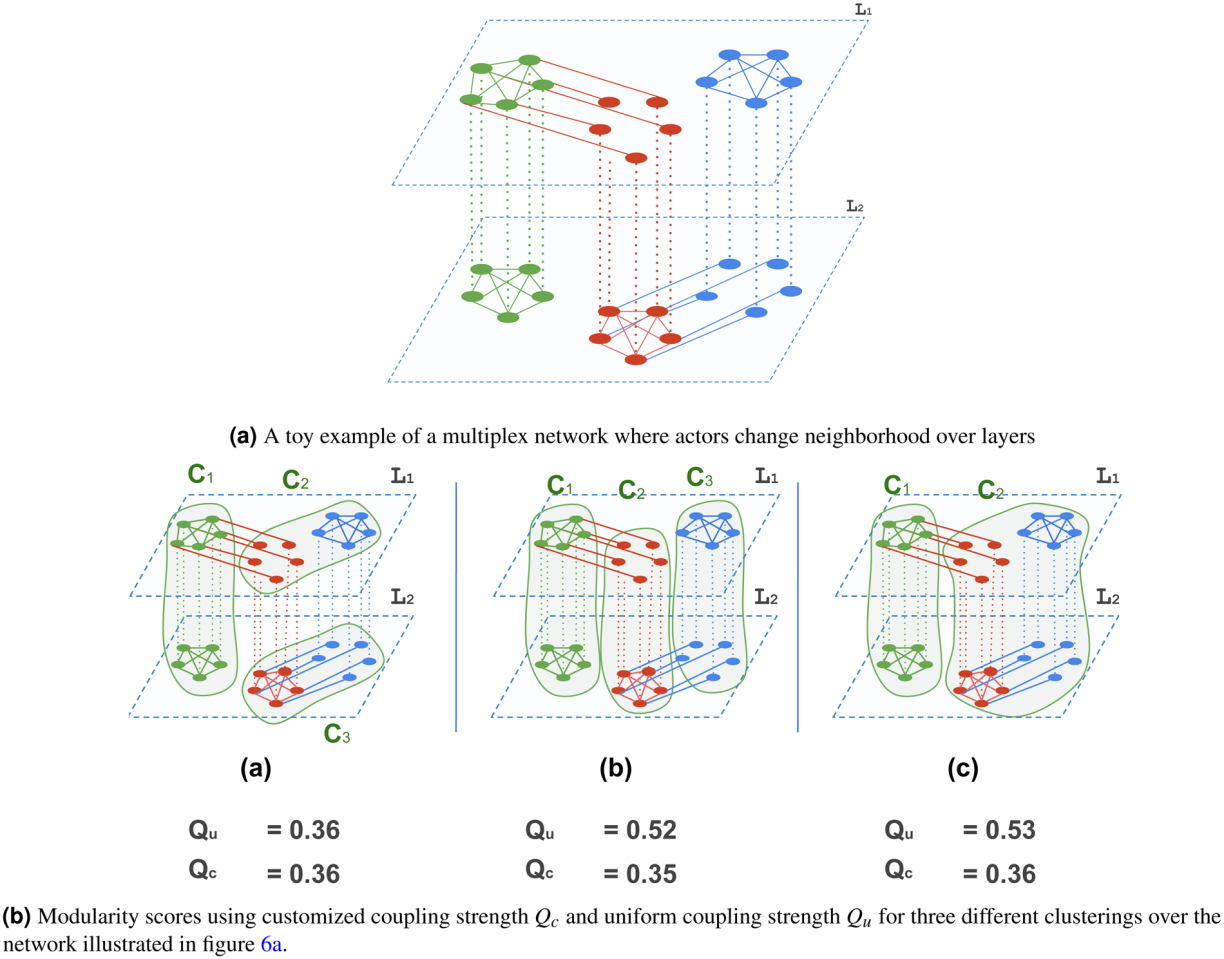
Effect of using customised coupling strength on the modularity scores.

In the paper introducing generalised modularity^21^, coupling edges were uniformly assigned to values greater than or equal to 0. Authors in^32^ suggested that coupling edges can convey more information and hence, they do not have to be uniformly assigned across layers. They can express the closeness among layers and hence closer layers are assigned larger values of $\omega$ and larger otherwise, and absent couplings among two layers should be expressed as couplings with a negative coupling strength ($\omega \, < \,0$). This allows the second part of Eq. 2 to penalise the co-existence of two nodes that refer to the same actor if the nodes are in two un-coupled layers. In^22^, the authors argued that couplings should be looked at from even a lower level, that is, the actor-level so nodes of an actor with similar neighbourhood over the layers should be maximally coupled (i.e., assigned a large $\omega$), while those with different neighborhoods across the layers should be minimally coupled. Figure 6b, for example, reports the multi-layer modularity scores using uniform coupling ${Q}_{u}$ with $\omega \,=\,1$ versus those using customised coupling ${Q}_{c}$ for three different clusterings over the network illustrated in Fig. 6a. The figure shows that assigning uniform coupling strength to all couplings might lead to favoring communities that expand over multiple layers even if actors nodes have different neighborhoods across the layers (${Q}_{u}$ is higher in cases (b) and (c)).

## Multi-layer Community Structures Recovered by Maximising Modularity

In this section, we will refer to the clustering of a single layer nodes resulted by maximising the modularity on that layer as the optimal single-layer groups, and to the clustering resulted by maximising the generalised modularity on the whole multi-layer network as multi-layer communities. We consider two groups of nodes in two different layers overlapping, if there exist at least two nodes (one in each group) that refer to the same actor. When two groups of nodes from two different layers appear together in one multi-layer community, we refer to that as cross-merging. We refer to the first part in Eq. 2 as the *intra-layer gain*, and to the second part as the *coupling gain*.

A multi-layer community detection task that maximizes multi-layer modularity involves partitioning the multi-layer network nodes into a clustering ${\mathcal{C}}$ that maximizes Eq. 2, as shown before. The intra-layer gain pushes nodes towards being partitioned into their optimal single-layer groups. The coupling gain pushes the overlapping optimal single-layer groups belonging to different layers towards cross-merging so to constitute multi-layer communities. The patterns identified by maximising both parts together largely depend on the value given to $\omega$ which seems to play the role of cross-merging orchestrator in the maximisation process (assuming that nodes are scanned in each layer separately first then across layers). For any solution not to partition the nodes according to their optimal single-layer groups, this results in a punishment in the intra-layer gain in Eq. 2. However, if this punishment can be compensated by the coupling gain, this gives some freedom (even if limited) to produce clusterings that do not firmly respect the single-layer optimal modularity constraints.

**Figure 7:**
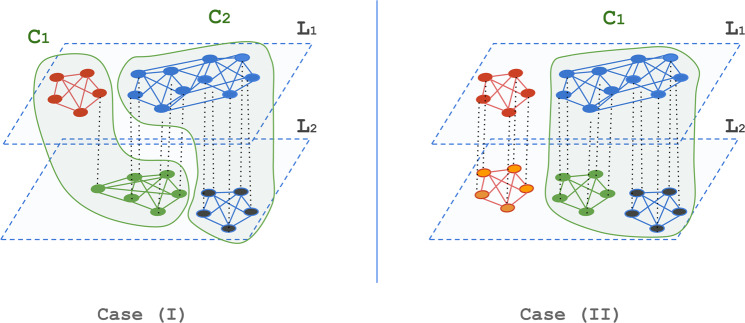
An illustration of two types of community structures possible to be recovered by modularity maximisation. Nodes are coloured according to their optimal single-layer grouping. Case (I), shows multi-layer communities that do not alter the optimal single-layer grouping. Case (II) shows a multi-layer community that alters the optimal single-layer grouping (i.e., it forces the blue and green nodes in layer ${L}_{2}$ to appear together in one community).

Let us assume that community labeling by multi-layer modularity maximisation happens in an order such that it scans the network nodes as follows. First, it scans pairs of nodes belonging to the same layer to maximise only the intra-layer gain, so nodes are labeled according to their optimal single-layer groups, and then it scans pairs of nodes belonging to different layers so their community labels can be updated such that the coupling gain is maximised. During the re-labeling phase, a node might face two types of relabeling: **(1)** the one that results only on a larger coupling gain without affecting the intra-layer gain. The result of this is cross-merging the overlapping optimal single-layer groups across layers without altering their single-layer grouping (i.e., without having to put two nodes that belong to two different single-layer groups in their layer together in the same multi-layer community). **(2)** One where adopting the new label requires the node to give up its optimal single-layer grouping and having to co-exist with nodes that do not belong to its optimal single-layer grouping. The result of this is cross-merging overlapping groups across layers and altering their single-layer grouping. Figure 7 illustrates the difference between the two cases where nodes are coloured according to their optimal single-layer grouping. As the figure shows, the multi-layer community ${C}_{2}$ in case (I) groups together nodes from both layers (the blue from layer ${L}_{1}$ and the black from layer ${L}_{2}$) without altering their optimal single-layer grouping. In case (II) however, the multi-layer community ${C}_{1}$ allows the grouping of the black and the green nodes from layer ${L}_{2}$ together even though in their optimal single-layer grouping they do not appear together.

**Figure 8:**
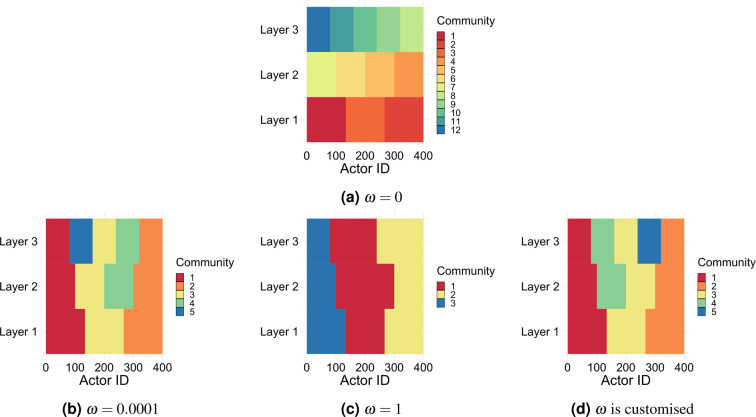
A coloured representation of the community assignment resulted by maximising the generalised modularity as a function of $\omega$ in a 3-layer 400-actor multiplex network. The x axes represent the actor id, while the y axes represent the layer, and different colours represent different community assignments.

Understanding the effect of $\omega$ on the resulted multi-layer community structure is very important for the interpretablity of these communities. In general, when the coupling style used to couple nodes across layers is adjacent or complete, low $\omega$ overstates the importance of the intra-layer edges and this results in multi-layer communities where nodes in each layer are in their optimal single-layer grouping (i.e., densely connected with each other in their layer and sparsely connected with the rest of the network). Assigning large values to $\omega$ washes out the effect of the intra-layer edges and overstates the importance of coupling edges (i.e., the co-existence of nodes belonging to the same actor in one community). This results in multi-layer communities where nodes do not necessarily fall in their optimal single-layer groupings. In both cases, providing an actor-level qualitative interpretation to such communities is not straightforward. Figure 8 provides a colored illustration of the grouping of nodes in a 3-layer 400-actor multiplex network for different assignments of $\omega$. The value $\omega \,\mathrm{=\; 0}$ shows the implanted grouping assigned to the nodes in each layer where each different color refers to a different group label. The multi-layer community structure resulted by maximising modularity is shown for $\omega \,\mathrm{=\; 0.0001}$, $\omega \,\mathrm{=\; 1}$ and when $\omega$ is customised based on the common neighbours across layers.

On a qualitative level, there are many cases where explaining multi-layer communities resulted by modularity maximisation is not an easy task. When the optimal single-layer groupings across layers are relatively aligned, the resulted multi-layer communities can be interpreted as groups of actors who interacted together significantly in all layers and less frequently with other actors out of their group. However, if the optimal single-layer groups are not aligned across layers, the interpretation of multi-layer communities resulted by modularity maximisation becomes less straightforward. One example can be the multi-layer communities in Fig. 8. Providing an actor-level interpretation to any community as “group of actors who….” such that this definition applies to all the other communities does not seem to be an easy task.

## Results

Here we report the results of testing the accuracy of modularity maximisation methods (i.e., ability to recover ground truth communities) for the different multi-layer community models proposed in Section 1. With each model, the accuracy is calculated as a function of the coupling strength ($\omega$) and the coupling style (adjacent or complete). To investigate whether coupling edges are treated as identity edges or as edges that connect nodes of the same community, we experimentally include a third coupling style, we refer to it as community-based, which couples nodes across layers only if they have the same community membership (given that the ground truth is known upfront and the generative model we use for our synthetic networks provides a node-level ground truth together with the generated networks). The following summarises our findings:

### From a multi-layer modularity perspective, inter-layer coupling edges are not perceived as identity edges but edges that couple nodes of the same community

**Figure 9:**
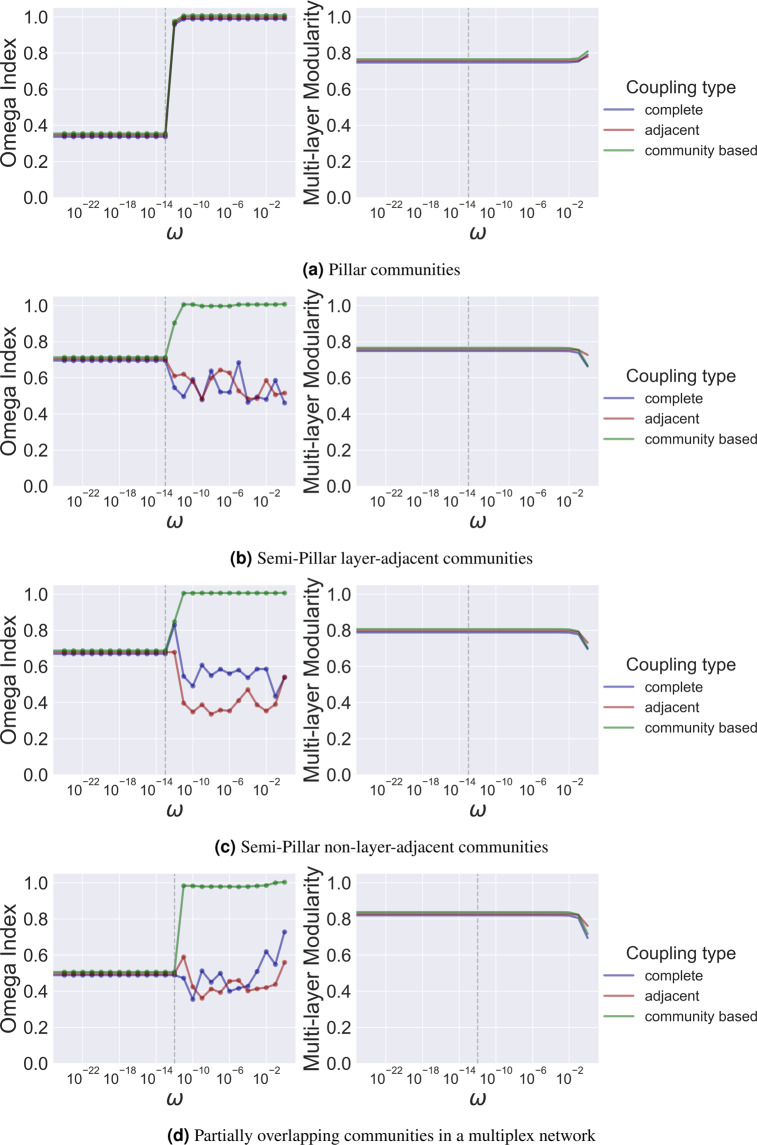
Accuracy using omega-index and modularity scores of community detection using multi-layer modularity maximisation as a function of the coupling type and the value of the coupling strength.

A general observation from our experiments (Fig. 9) is that the highest accuracy of community discovery with more complex community models than the pillar model is achieved only when the coupling style is community-based (only nodes of the same community are coupled). This suggests that multi-layer modularity maximisation treats inter-layer coupling edges as community edges and when coupling-edges are used as identity-edges (complete or adjacent), the accuracy of modularity maximisation seems to drop down with more complex community models than the pillar one.

### Less accurate does not necessarily imply less modular according to multi-layer modularity

While the accuracy scores fluctuate as a function of both the coupling style and the value of the coupling strength (Fig. 9), modularity scores seem to be stable. This suggests that less accurate clusterings are not necessarily less modular according to the generalised modularity.

### Pillar communities are accurately recoverable under all coupling styles and a very small positive assignment of *ω*

As shown in Fig. 9a, a very small positive assignment of $\omega$ ($\omega ={10}^{-13}$) set uniformly across layers guarantees a very high accuracy for recovering pillar communities independently of the coupling style used to couple nodes across layers.

### Semi-pillar communities and partially overlapping communities cannot be accurately recovered using adjacent or complete coupling styles and a uniform positive assignment of *ω*

As the community model gets more complex compared to the pillar model, modularity maximisation seems to fail at accurately recovering the ground truth communities when the coupling style is the adjacent or the complete one independently of the value given to the coupling strength $\omega$ (Fig. 9b–d). However, if coupling edges are placed only among nodes belonging to the same multi-layer community, multi-layer modularity maximisation seems to be able to recover the ground truth communities and shows a stable behaviour with respect to the value given to $\omega$.

### With hierarchical communities, the value *ω* controls the granularity of community detection

**Figure 10:**
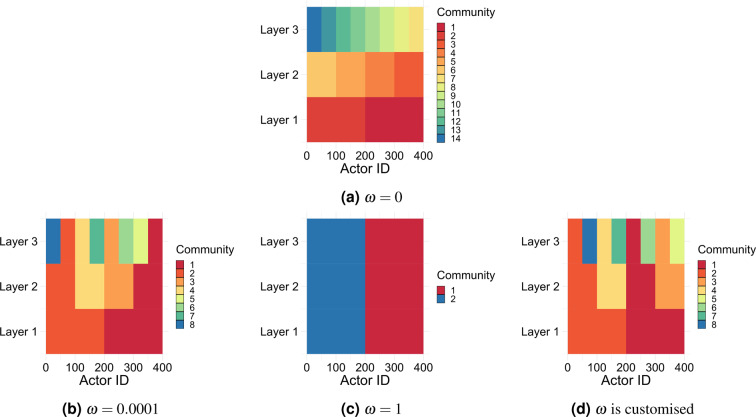
A coloured representation of the community assignment resulted by maximising the generalised modularity as a function of $\omega$ in a 3-layer 400-actor multiplex network. The x axes represent the actor id, while the y axes represent the layer, and different colours represent different community assignments.

With the hierarchical community model (M5), we study the structure of the detected communities as a function of $\omega$, instead of reporting their accuracy. Figure 10b–d illustrate the structure of the detected multi-layer communities using modularity maximisation when the implanted communities in each layer reflect the level of hierarchy illustrated in Fig. 10a. We show only the results when the coupling type is complete since the other types do not significantly change the output in this model. We can see that using high values of $\omega$ forces the community membership of the highest level in the hierarchy across the layers. Nonetheless, using lower values of $\omega$ does not force the community membership of the highest level of the hierarchy across all the nodes across the layers but only part of them such that the single-layer optimal groupings are guaranteed.

## Discussion

In this article, we investigated multi-layer modularity maximisation, an extensively used tool for community discovery in multi-layer networks. We infer unspoken assumptions that condition the ability of multi-layer modularity maximisation to recover ground truth communities by investigating the role of $\omega$ and coupling edges introduced in multi-layer modularity. Our main findings can be summarised as follows: ***(1)*** the high accuracy of multi-layer modularity maximisation is conditioned by a coupling style that couples only nodes of the same community together, ***(2)*** when a proper coupling is chosen in a multi-layer network generated using one of the models (M1–M4),- the value of $\omega$ assigned to the coupling edges has no impact on the accuracy of community detection using multi-layer modularity maximisation (as long as $\omega  > \mathrm{0)}$, ***(3)*** less accurate does not necessarily mean less modular according to multi-layer modularity, and ***(4)*** with hierarchical multi-layer communities, $\omega$ controls the granularity of the detected patterns and the qualitative interpretation of the identified patterns is not always clear.

The fact that the accuracy of multi-layer modularity maximisation with more complex multi-layer community models is conditioned by coupling nodes of the same community together (rather than the same actor) sheds a light on the importance of coupling style for a successful recovery of the communities and hints to limitations in the coupling strategies available in the literature to couple nodes in multi-layer networks. While some heuristics can be developed to predict whether two nodes of an actor belong to the same community or not, and a community-based coupling can be built on top of that, a valid question can be: why should the accurate recovery of multi-layer communities be conditioned by knowing which nodes of the same actor fall in the same community, which is what the community detection method itself is supposed to infer?

The reason why networks should be analysed in a multi-layer manner is that there are dependencies among different interactions/relationships and providing any analysis by considering only one of these interactions might give misleading or incomplete findings. This means that for the formation of multi-layer communities, different dependencies among the layers might result in different structures of multi-layer communities (a very high dependency among all layers for example results in pillar communities). While this motivation is sound, the question becomes whether it is always possible to reflect these inter-dependencies in the tools used for multi-layer network analysis, especially community detection. Thinking of multi-layer modularity, the coupling strength *ω* (as suggested by the original authors^21^) is a binary variable that takes one of the two values: either 0 to refer to the non-existence of a coupling edge between two nodes of an actor, or a positive value *ω* > 0 to refer to the existence of that coupling edge. When the proper coupling is provided, our experiments showed that the positive value assigned to the existent couplings does not affect the accuracy of community detection. This means that this parameter with models (M1-M4) has no role more than referring to existent (*ω* > 0) or non-existent (*ω* < = 0) couplings. This adds another limitation to modularity maximisation based methods because with this limited interpretation of *ω*, the only multi-layer ingredient in the generalised modularity, there is no way to reflect different levels of inter-dependencies among the different layers and/or nodes of an actor and to take these inter-dependencies into account in community discovery.

As shown in our experiments, multi-layer modularity scores do not necessarily follow the same trends the accuracy of the recovered communities follow. Indeed, the modularity scores of the accurately recovered clusterings are not necessarily higher than those with lower accuracy. This raises another important question: is multi-layer modularity maximisation a valid proxy for finding complex multi-layer communities? Or is the idea of it being as such mostly inherited from the success and the popularity the single-layer implementation has had?

The qualitative interpretation of the recovered patterns in networks generated using one of the community models (M1–M4) does not change much from those identified using the single-layer modularity. If edges were created such that nodes of the same community are densely connected within the community and sparsely connected with the rest of the network, the patterns identified by maximising multi-layer modularity satisfy that condition. With the hierarchical community model, however, it is not clear how to interpret the identified patterns for values of *ω* other than 0 (the lowest granularity of the hierarchy). The question is whether using multi-layer community detection in this case provides any additional information the single-layer community detection on each layer separately cannot provide and we tend to believe that multi-layer community detection here using multi-layer modularity maximisation can give misleading results from a qualitative perspective.

## Methods

For recovering the multi-layer communities that maximise multi-layer modularity, we chose Generalised Louvain method^33^ as a representative method for the class of modularity maximisation community detection methods. The choice of this method comes for both being a well-referenced method in the literature and serving as one of the best approximation methods among other modularity-maximisation methods in terms of its accuracy and performance. Since communities resulted by this method might vary from one execution to another because the order by which the nodes are scanned by this method is chosen at random, we provide the final result of community detection after 10 executions and choosing the one with the maximum modularity as an output.

For each of the community models (M1–M4) we calculate the accuracy of the resulted communities as a function of the coupling style (adjacent, complete, and community based) and the value of the coupling strength *ω* assigned to the coupling edges. For measuring accuracy, we chose omega-index for its sensitivity to different types of dissimilarities among clusterings and its ability to remove the by-chance agreement from the final score as we discussed in a previous study^34^.

As regards the generation of the multi-layer networks, we refer to the generative model in^16^. To the best of our knowledge, this provides the most general platform in the literature for generating synthetic multi-layer communities that takes into consideration different types of multi-layer networks. The generation of a multi-layer community using this model goes through the following three steps. First, assuming a multi-layer network of n actors and m layers and initial number of communities *k* to be planted in each layer, the model starts by assigning nodes in each layer randomly to *k* community memberships (a categorical distribution can be used for this step). At the end of this phase, nodes in each layer will be distributed over *k* not necessarily equal in size groups. In the second phase, nodes start to propagate their community memberships across layers with a probability equal to the dependency between the layers. The type of multi-layer network we want to generate, i.e., multiplex or time-dependent, controls the order and the way this propagation of community labels across layers happen. At the end of this phase, community memberships that were set in the first phase might be updated based on the assumed dependency p set among layers. At the third phase, a multi-layer edge generation model can be used to create edges of the multi-layer network given the implanted community assignments resulted from the previous step. In our experiments, we generate out synthetic networks using a variant of the degree-corrected SBM benchmark that avoid the creation of self-loops and parallel edges^16^. We chose to fix the mixing parameter in edge generation phase in our experiments to a very small value ($\mu \,\mathrm{=\; 0.05}$). That is because our main goal from the experiments is not to test the ability of multi-layer modularity maximisation to recover noisy patterns, but to test the ability to recover complex multi-layer patterns.

We chose our synthetic multi-layer networks to be 4-layer networks of 1000 actors (i.e., 4000 nodes). To generate pillar communities (M1), we use the aforementioned generative model and we generate a temporal network with a very high dependency across the layers (p = 1). To generate the semi-pillar layer-adjacent communities (M2), we generate two sets of pillar partitions on two 2-layer temporal networks. We use the resulted partitions to generate a 4-layer multi-layer network where in the first two layers we plant the first set of partitions and in the last two layers we plant the second set of partitions. For (M3), we do the same except that the 4-layer multi-layer network is resulted by implanting the first set of partitions in the first and the third layer, while in the second and the fourth we plant the second set of partitions. As to partially overlapping communities, we generate multiplex networks with a moderate dependency among layers (p = 0.3). This, according to the used generative model, results in communities that are not perfectly pillar but partially overlapping.
